# Martensitic transformation of FeNi nanofilm induced by interfacial stress generated in FeNi/V nanomultilayered structure

**DOI:** 10.1186/1556-276X-9-440

**Published:** 2014-08-27

**Authors:** Wei Li, Ping Liu, Ke Zhang, Fengcang Ma, Xinkuan Liu, Xiaohong Chen, Daihua He

**Affiliations:** 1 School of Materials Science and Engineering, University of Shanghai for Science and Technology, Shanghai 200093, China

**Keywords:** FeNi alloy, Multilayer thin films, Martensitic phase transformation, Interfacial stress, Epitaxial growth

## Abstract

FeNi/V nanomultilayered films with different V layer thicknesses were synthesized by magnetron sputtering. By adjusting the thickness of the V layer, different interfacial compressive stress were imposed on FeNi layers and the effect of interfacial stress on martensitic transformation of the FeNi film was investigated. Without insertion of V layers, the FeNi film exhibits a face-centered cubic (fcc) structure. With the thickness of V inserted layers up to 1.5 nm, under the coherent growth structure in FeNi/V nanomultilayered films, FeNi layers bear interfacial compressive stress due to the larger lattice parameter relative to V, which induces the martensitic transformation of the FeNi film. As the V layer thickness increases to 2.0 nm, V layers cannot keep the coherent growth structure with FeNi layers, leading to the disappearance of interfacial compressive stress and termination of the martensitic transformation in the FeNi film. The interfacial compressive stress-induced martensitic transformation of the FeNi nanofilm is verified through experiment. The method of imposing and modulating the interfacial stress through the epitaxial growth structure in the nanomultilayered films should be noticed and utilized.

## Background

Martensitic transformation in nanostructured materials has attracted considerable scientific interest over the past decades because phase transformation behaviors in nanostructured materials are different from their conventional coarse-grained counterparts [1,2]. To explain abnormal martensitic transformation behaviors, many competing theories have been suggested, including lack of nucleation sites [3], inhibition of nanotwin boundaries [4], surface energy difference [5,6], and interfacial stress due to particle curvature [7,8]. Among them, it is widely believed that interfacial stress plays an important role in abnormal martensitic transformation of nanostructured materials due to the high volume fraction of interfaces. Nevertheless, this viewpoint has only been brought forward in theories, which has difficulty to be verified through experiment. In addition, stress-induced martensitic transformation has been widely observed and investigated in past half a century [9-11]. Martensitic transformations could be found to be affected in a variety of ways of the application of stress. However, whether the martensitic transformations in nanostructured materials can be influenced by the nanoscaled stress has rarely been documented, which is of great importance to martensitic transformation research in nanostructured materials.

The above investigations are difficult to carry out owing to the fact that it is difficult to artificially impose the nanoscaled stress within nanostructured materials. Fortunately, the current studies on nanomultilayered films provide us a feasibility of artificially imposing the interfacial stress in the nanosized films. Through alternately depositing two layers with different lattice parameters, *d*, the two layers can bear the interfacial tensile or compressive stress under the coherent growth structure in nanomultilayered films [12,13]. Furthermore, the interfacial stress can be modulated by changing the modulation period and ratio of two layers. To this end, Fe_50_Ni_50_ alloy (at.%, face-centered cubic (fcc) structure, *d* is 342 pm [14] (1 pm = 10^-12^ m)) with typical martensitic transformation [15,16] and V (body-centered cubic (bcc) structure, *d* is 302.4 pm) without allotropic transformation are alternately deposited to synthesize FeNi/V nanomultilayered films. By altering the thickness of the V layer, different interfacial stress will be imposed on FeNi nanolayers under the coherent growth structure and the effect of interfacial stress on martensitic transformation of the FeNi nanofilm will be investigated.

## Methods

### Materials

The FeNi/V nanomultilayered films were fabricated on silicon substrates by a magnetron sputtering system. The FeNi layer was deposited from a Fe_50_Ni_50_ alloy target (at.%, 99.99%) by DC mode, and the power was set at 100 W. The V layer was sputtered from a V target (99.99%) by RF mode, and the power was set at 80 W. Both FeNi and V targets were 75 mm in diameter. The substrates were ultrasonically cleaned in acetone and alcohol before being mounted on a rotatable substrate holder in the vacuum chamber. The distance between the substrate and target was 50 mm. The base pressure was pumped down to 5.0 × 10^-4^ Pa before deposition. The Ar flow rate was 15 sccm. The working pressure was 0.4 Pa, and the substrate was heated up to 300°C during deposition. The configuration of FeNi/V nanomultilayered films was designed with FeNi layers with a fixed thickness of about 10 nm along with variable V layer thickness ranging from 0.5 to 3.0 nm. The individual modulation layer thickness of the multilayered film was obtained by controlling the staying time of the substrates in front of each target. The monolithic FeNi film (without insertion of V nanolayers) was also fabricated for comparison. The thickness of all films was about 2 μm.

### Characterization

The microstructures of FeNi/V nanomultilayered films were investigated by X-ray diffraction (XRD) using Bruker D8 Advance (Bruker AXS, Inc., Madison, WI, USA) with Cu K_a_ radiation and field emission high-resolution transmission electron microscopy (HRTEM) using Philips CM200-FEG (Philips, Amsterdam, The Netherlands). The composition was characterized by an energy-dispersive spectroscopy (EDS) accessory equipped in a Philips Quanta FEG450 scanning electron microscope (SEM). The XRD measurements were performed by a Bragg-Brentano (*θ*/2*θ*) scan mode with the operating parameters of 30 kV and 20 mA. The diffraction angle (2*θ*) range for high-angle diffraction pattern was scanned from 40° to 70°. The preparation procedures of the cross-sectional specimen for TEM observation are as follows. The films with a substrate were cut into two pieces and adhered face to face, which were subsequently cut at the joint position to make a slice. The slices were thinned by mechanical polishing followed by argon ion milling.

## Results and discussion

**Figure 1:**
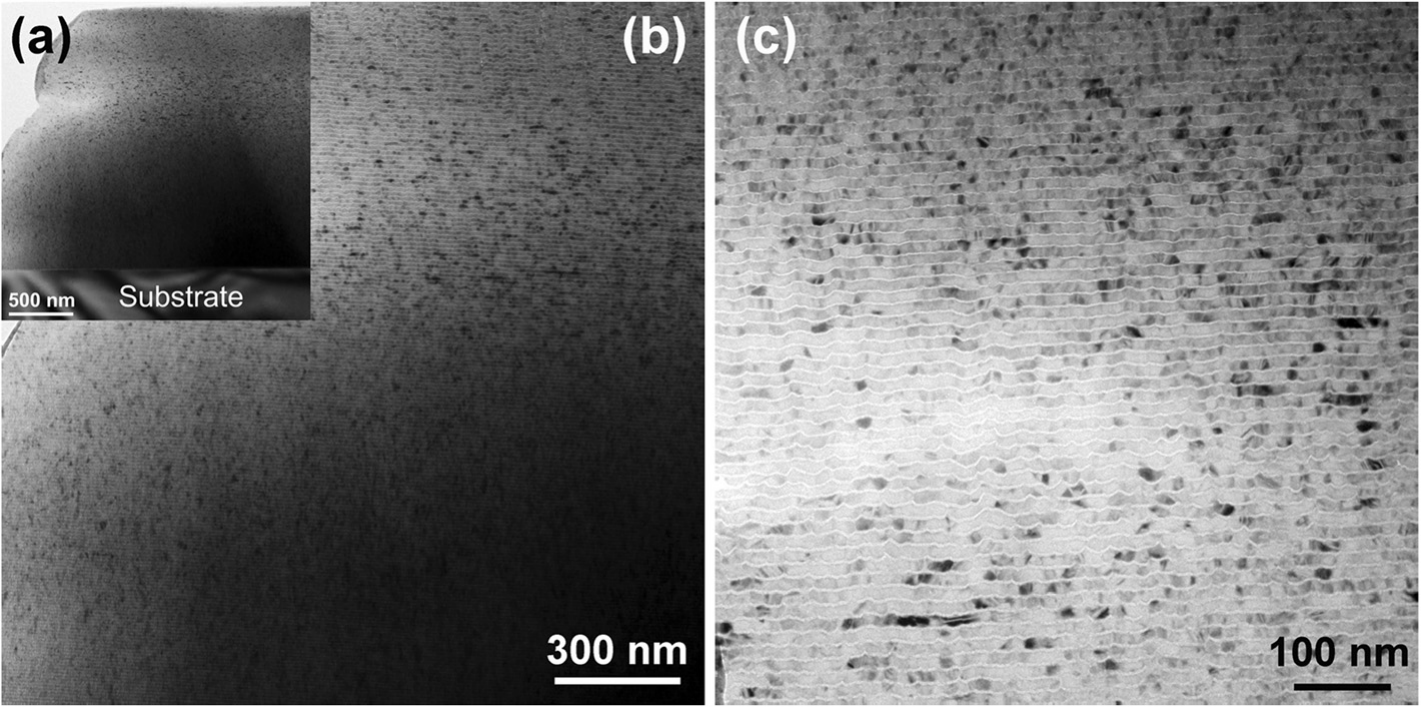
**Cross-sectional HRTEM images of the FeNi/V nanomultilayered film with V layers deposited for 6 s. (a)** Low magnification. **(b)** Medium magnification. **(c)** High magnification.

Figure 1 shows the typical cross-sectional HRTEM images of the FeNi/V nanomultilayered film with V layers deposited for 6 s. From the low-magnification image of Figure 1a, it can be seen that the FeNi/V nanomultilayered film presents a compact structure and smooth surface, with the thickness of about 2.0 μm. Figure 1b exhibits that the FeNi/V nanomultilayered film is composed of a microscopic multilayered structure. It is clear from the magnified Figure 1c that FeNi and V layers form an evident multilayered structure with distinct interfaces. The thick layers with dark contrast and thin layers with bright contrast correspond to FeNi and V, respectively.

**Figure 2:**
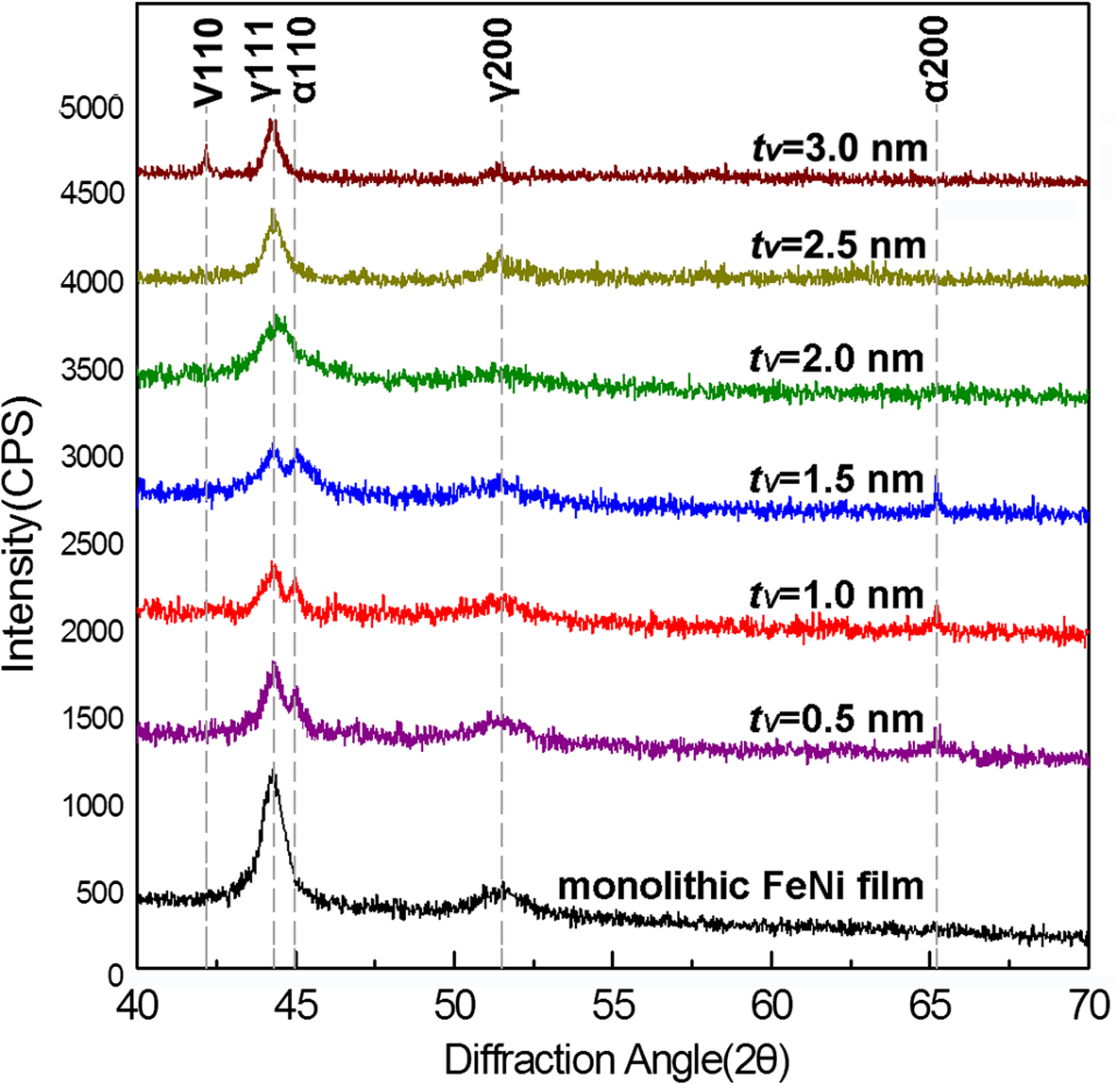
XRD patterns of the monolithic FeNi film and FeNi/V nanomultilayered films with different V layer thicknesses.

The XRD patterns of the monolithic FeNi film and FeNi/V nanomultilayered films with different V layer thicknesses (*t*_V_) are shown in Figure 2. It is worth noting that, from the EDS results, the composition (at.%) of the monolithic FeNi film is 49.56% Fe and 50.44% Ni, which is basically consistent with that of the Fe_50_Ni_50_ (at.%) alloy target. The composition of the FeNi layer in the FeNi/V nanomultilayered film is consistent with that of the monolithic FeNi film because both films were prepared by the same Fe_50_Ni_50_ (at.%) alloy target. It can be seen that the monolithic FeNi film exhibits a fcc structure (γ), without existence of martensite (α) with a bcc structure. With the initial increase of V layer thickness, bcc-structured FeNi phase is detected in nanomultilayered films besides fcc-structured FeNi phase, suggesting that martensitic transformation occurs in FeNi layers. In addition, with the increase of deposited time from 2 to 6 s, the diffraction peaks for fcc-structured FeNi weaken, while those for bcc-structured FeNi strengthen. According to the deposition rate of V (about 0.25 nm/s) derived from the monolithic V film, the thicknesses of the V layers deposited for 2, 4, 6, 8, 10, and 12 s at the same condition are 0.5, 1.0, 1.5, 2.0, 2.5, and 3.0 nm, respectively, which have been indexed in the corresponding XRD patterns in Figure 2. When the V layer thickness increases from 1.5 to 2.0 nm, however, the bcc-structured FeNi can hardly be detected, implying that the martensitic transformation of FeNi terminates. As the V layer thickness further rises to 3.0 nm, the (110) diffraction peak of bcc-structured V emerges in the XRD patterns besides fcc-structured FeNi, suggesting that V layers begin to present a stable bcc structure.

**Figure 3:**
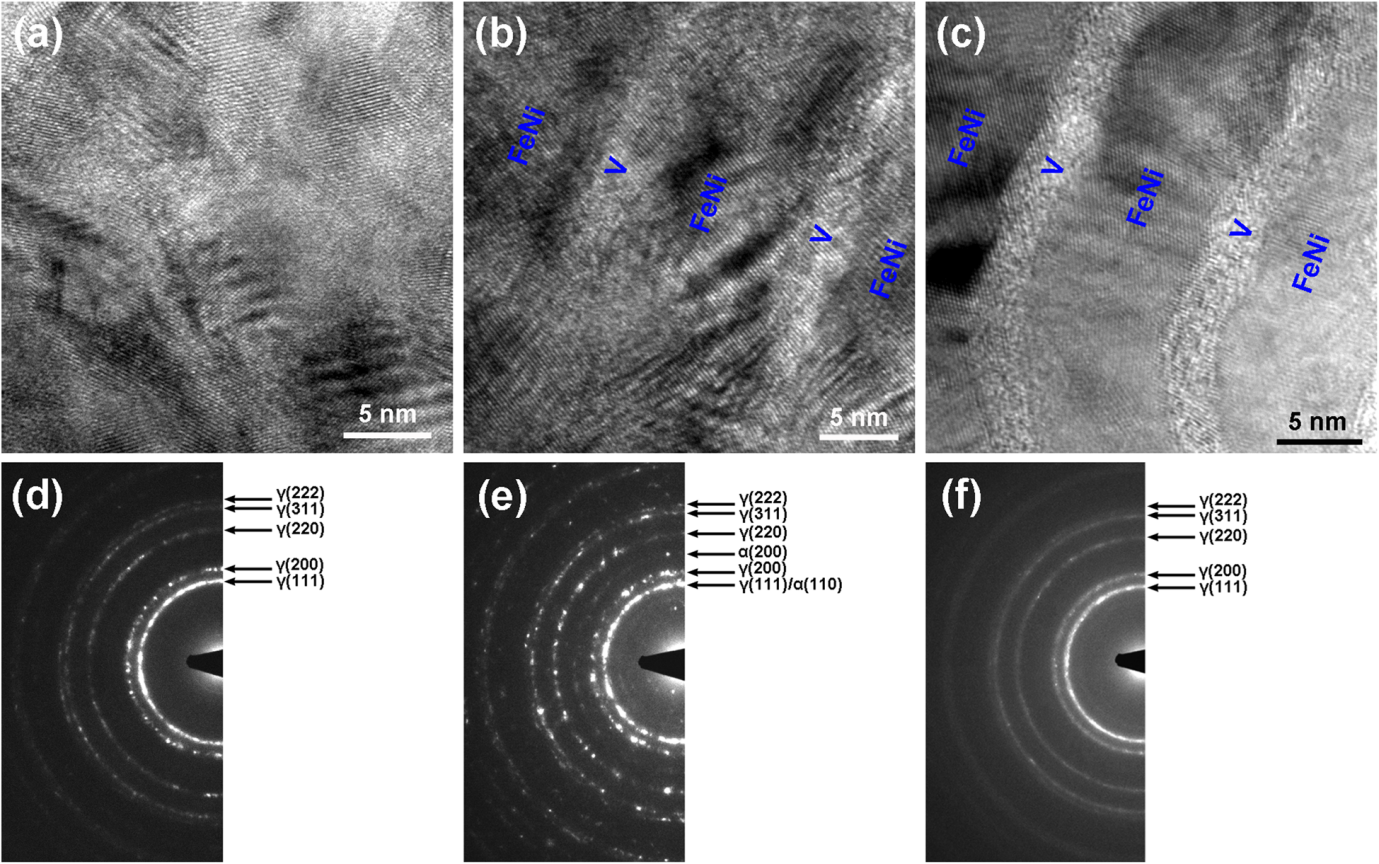
**Cross-sectional HRTEM images and selected area electron diffraction (SAED) patterns. (a, d)** Monolithic FeNi film and FeNi/V nanomultilayered films with V layer thicknesses of **(b, e)** 1.5 nm and **(c, f)** 2.0 nm.

According to the investigation of nanomultilayered films, when two crystallized layers form a nanomultilayered film by alternate deposition, if the thickness of one layer is small enough, this layer will transform into the same structure with the other and grow epitaxially with the other, in order to lower the interfacial energy of the whole film system [17], such as TiN/AlN [18], TiB_2_/VC [19], and ZrO_2_/TiN [20] nanomultilayered films. Under the epitaxial growth structure formed in the nanomultilayered films, the originally larger lattice parameter of one layer is inclined to decrease, leading to generation of interfacial compressive stress, while the originally smaller lattice parameter of the other layer is forced to increase, resulting in formation of interfacial tensile stress. In the FeNi/V nanomultilayered films, due to the small thickness of V layers, the bcc-structured V layers can be forced to transform into a fcc structure and grow epitaxially with the FeNi layers. The lattice parameters for Fe_50_Ni_50_ and V, respectively, are 342 and 302 pm. Under the epitaxial growth structure, FeNi layers will bear the interfacial compressive stress. Therefore, it can be deduced that the martensitic transformation of FeNi layers can be induced by interfacial compressive stress within the FeNi/V nanomultilayered films. When the thickness of the V layer further increases to 2.0 nm, V layers cannot maintain the epitaxial growth with the FeNi layers, leading to disappearance of interfacial stress and termination of the martensitic transformation in the FeNi film. Nevertheless, the epitaxial growth structure and its induced martensitic transformation need to be further verified from HRTEM investigation.Figure 3 presents that the typical cross-sectional HRTEM images and selected area electron diffraction (SAED) patterns of the monolithic FeNi film and FeNi/V nanomultilayered films with different V layer thicknesses. It can be observed from Figure 3a that atomic arrangement in the monolithic FeNi film has high periodicity, indicating that the film is well crystallized. The SAED pattern in Figure 3d shows that the monolithic FeNi film only exhibits a fcc structure, which is consistent with the XRD result. From Figure 3b, it can be seen that the dark and bright layers, corresponding to FeNi and V, respectively, are about 10 and 1.5 nm, which are consistent with the structure design. As the V layers with the thickness of 1.5 nm are inserted in the FeNi film, the lattice fringes continuously go through several layers and interfaces, indicating that V layers have not existed in a bcc structure, but transformed to a fcc structure and grown epitaxially with FeNi layers, which validates the above deduction from the XRD results. From the SAED pattern in Figure 3e, the film is composed of both fcc and bcc structures. According to the above analysis and XRD results, the bcc-structured phase corresponds to FeNi, rather than V. Therefore, it can be reasonably believed that the martensitic transformation occurs in the FeNi layers of the FeNi/V nanomultilayered film under the epitaxial growth structure between FeNi and V layers. As the V layer thickness increases to 2.0 nm, however, V layers cannot maintain the epitaxial growth with FeNi layers, but present an amorphous state, as shown in Figure 3c. The lattice fringes in FeNi layers cannot traverse through the V layers, manifesting the epitaxial growth structure is blocked by the V layers. The SAED pattern in Figure 3f indicates that only a fcc structure exists within the film, suggesting that martensitic transformation in FeNi layers terminates, which agrees with the XRD results.

It is worth noting that the diffraction information of V layers is not detected in the SAED patterns for the FeNi/V nanomultilayered films with different V layer thicknesses in Figure 3, which can be attributed to two aspects. Firstly, when V layers grow epitaxially with FeNi layers, V layers transform into a fcc structure under the template effect of FeNi layers, and the lattice parameter is inclined to increase and approach that of FeNi. Therefore, the SAED rings of V may coincide with those of FeNi. A similar phenomenon could also be found in our recent investigation of CrAlN/ZrO_2_ nanomultilayered films [21]. When the thickness of the ZrO_2_ layer was less than 1.0 nm, the originally tetragonal-structured ZrO_2_ layers were forced to transform to a pseudomorphic fcc structure and grew epitaxially with CrAlN layers. In this case, the SAED patterns can be only composed of a fcc structure, without detection of a tetragonal structure. Secondly, as the V layer thickness increases to 2.0 nm, amorphization can be the reason of the absence of diffraction information of V layers in the SAED patterns. With the thickening of V layers, V gradually transforms from the metastable fcc structure to a stable bcc structure due to the difference of strain-free bulk energy [22]. The amorphization can be the transition state between the fcc structure and bcc structure. From the XRD results, V layers transform from the transient amorphous state into a stable bcc structure when the V layer thickness increases to 3.0 nm. Therefore, when the V layer thickness is in the range of 2.0 ~ 3.0 nm, V layers present the amorphous state between fcc structure and bcc structure. We also observed the amorphization of yttrium (Y) layers between fcc structure and hcp structure with the increase of Y layer thickness in FeNi/Y nanomultilayered films, which will be discussed in another paper. It must be pointed out that amorphous-featured diffraction corona is not observed in the SAED pattern, which can be attributed to the facts that the diffraction information is only gathered from the circular region with the diameter of about 20 nm and in such small area, the low amount V with the thickness of 1.5 nm cannot produce enough strong diffraction signal.

The microstructural evolution of V layers in FeNi/V nanomultilayered films can be explained by a thermodynamic model. The total energy of the V layer, *E*_T_, is composed of strain-free bulk energy, strain energy, and interfacial energy, which can be written as

(1) $$E_{T}=\left(E_{\text{bulk}}+E_{\text{str}}\right)t_{V}+E_{\text{int}}$$

where *E*_bulk_ and *E*_str_, respectively, are the strain-free bulk energy and strain energy per unit of V layer, in which *E*_str_ takes a larger value with a small *t*_V_ and decreases with the increase of *t*_V_, and *E*_int_ is the interfacial energy between FeNi and V layers.

**Figure 4:**
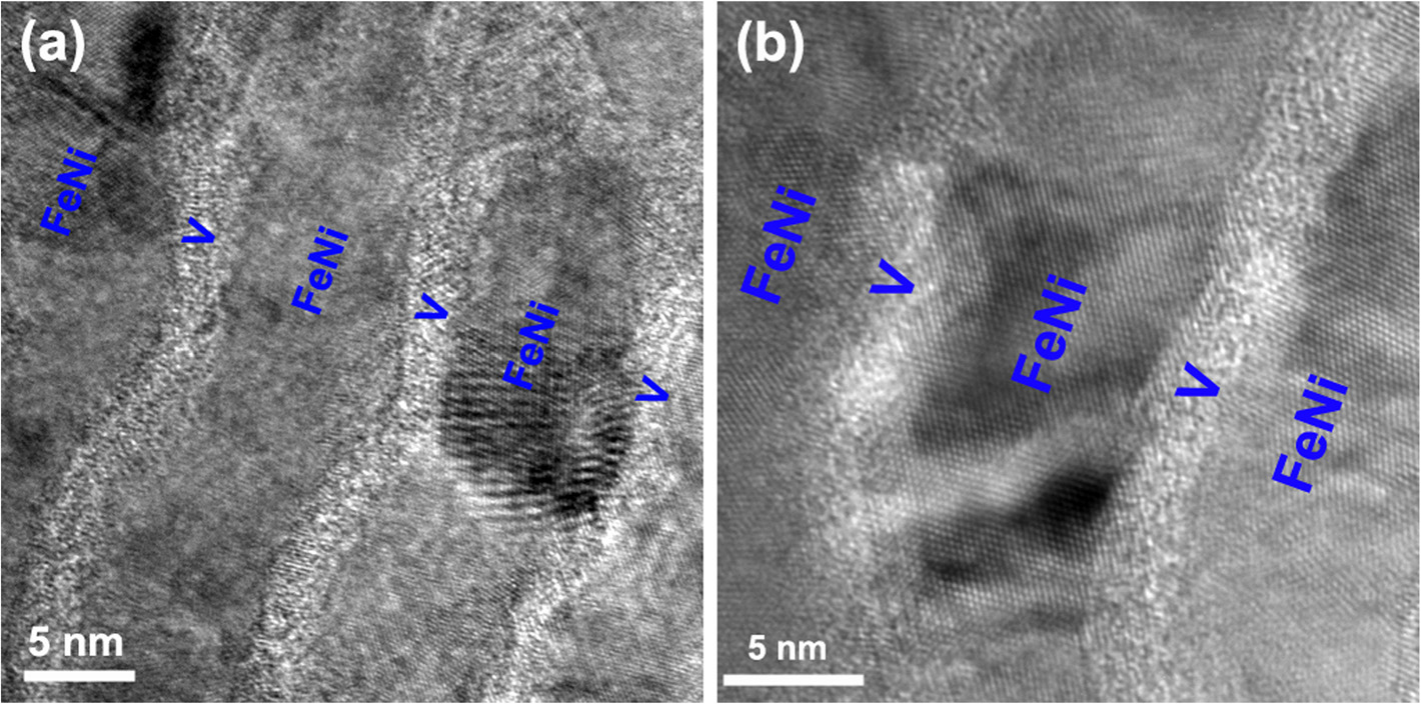
**Amorphization of V layers within the FeNi/V nanomultilayered film with a V layer thickness of 2.0 nm. (a)** Low magnification. **(b)** High magnification.

During the initial increase of *t*_V_ (less than 1.5 nm), since *t*_V_ is small, *E*_int_ is the main component of *E*_T_. Formation of a coherent interface between FeNi and V layers can lower *E*_int_. Therefore, V layers can transform into a fcc structure and grow epitaxially with FeNi layers. When *t*_V_ rises to 2.0 nm, the strain-free bulk energy and strain energy increase, which occupy a larger proportion in *E*_T_ than in *E*_int_. *E*_T_ cannot be reduced by forming the coherent interface. Therefore, the V layers cannot maintain the fcc structure and epitaxial growth with FeNi layers. In addition, since *E*_str_ takes a larger value when *t*_V_ is comparatively small, *E*_T_ is dominated by the strain energy relative to the strain-free bulk energy. In this situation, formation of a bcc structure of V layers within the FeNi/V nanomultilayered film can lead to the increase of the strain energy. Consequently, amorphization, as a transition state between fcc and bcc structures, has been formed to lower the strain energy and thus *E*_T_, as additionally shown in Figure 4.

**Figure 5:**
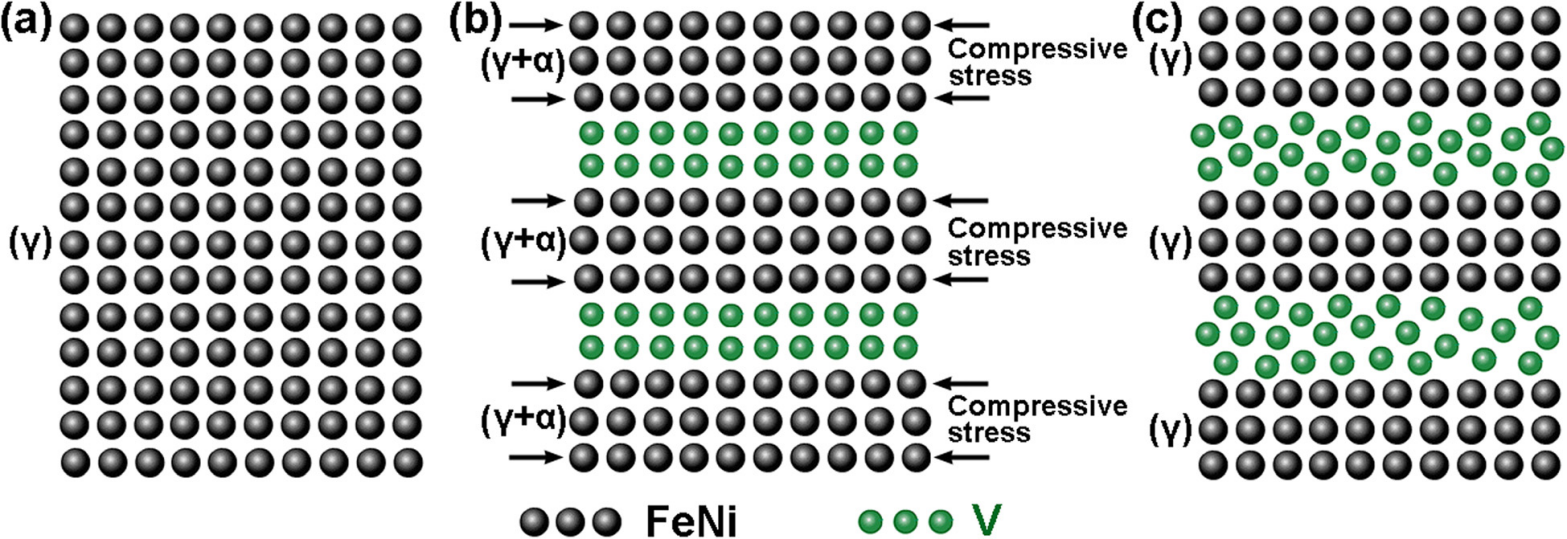
**Schematic illustration of the microstructural evolution of FeNi/V nanomultilayered films with different V layer thicknesses. (a)** Without insertion of V layers. **(b)** Less than 1.5 nm. **(c)** 2.0 nm.

The microstructural evolution of the FeNi/V nanomultilayered films with the increase of V layer thickness is illustrated in Figure 5. As shown in Figure 5a, without insertion of V layers, the FeNi film exhibits a fcc structure. When the thickness of V inserted layers is less than 1.5 nm, V inserted layers can transform into a fcc structure under the template effect of FeNi layers and grown epitaxially with FeNi, as indicated in Figure 5b. Since the lattice parameter of V is smaller than that of FeNi, under the coherent growth structure, FeNi layers bear interfacial compressive stress generated from V layers. In reference to the alternating-stress field strengthening theory [23], the maximum shear stress on the interfaces could be calculated as Equation 2:

(2) $$\tau_{\text{max}}=\frac{\sqrt{6}}{6}A\cdot E_{\mathrm{WA}}\cdot \eta$$

where *A* is the modulation amplifying factor influenced by modulation period, modulation ratio, and roughness and width of interfaces. According to the studies from Mirkarimi [24] and Shinn [25], *A* takes the value of 0.5 for calculation in this investigation. *E*_WA_ is the weighted average elastic modulus of the bilayer layers, which is calculated as 195.8 GPa for a FeNi(10 nm)/V(1.5 nm) nanomultilayered film based on the elastic modulus values for Fe_50_Ni_50_ (206 GPa) and V (128 GPa). *η* is the lattice mismatch between two layers of multilayers. Since V layers transform into a fcc structure, it is difficult to calculate the lattice mismatch between two layers. If it is assumed that the lattice mismatch is between 3% and 5%, the maximum shear stress is about 1.20 to 1.99 GPa according to Equation 2.

Stress-induced martensitic transformation has been widely observed and investigated in past decades. Hsu and his collaborators successfully predicted the start temperatures of martensitic transformation (*M*_*s*_) in Fe-C, Fe-X, and Fe-X-C alloys by the thermodynamics theories and believed that applied stress, as a driving force, could promote martensitic transformation and thus elevate *M*_*s*_[26-29]. Gautier et al. reported a linear enhancement of *M*_*s*_ in Fe-Ni alloys with applied stress (*σ*) with d*M*_*S*_/d*σ* of 0.07°C/MPa for a cooling rate of 0.5°C/s [30]. According to this result, *M*_*s*_ of the FeNi layer in the FeNi/V nanomultilayered film should increase from 84°C to 139.3°C relative to that with no interfacial stress. Therefore, interfacial compressive stress generated in the nanomultilayered film can induce martensitic transformation of the FeNi layer.As the thickness of V layers increases to 2.0 nm, as shown in Figure 5c, V layers can hardly keep their fcc structure, and transform into an amorphous state, which destroys the coherent growth structure, leading to the appearance of interfacial compressive stress. Accordingly, the martensitic transformation in FeNi layers terminates, and the FeNi layers transform back into the original fcc structure.

In this investigation, it is experimentally confirmed that interfacial compressive stress in nanoscale can induce the martensitic transformation in FeNi nanolayers. Generally, within the nanostructured materials, a large amount of interfacial stress could exist owing to the high volume fraction of interfaces, which might modulate the martensitic transformation of the nanostructured materials and make the martensitic transformation behaviors in the nanostructured materials differ from their conventional coarse-grained counterparts. Utilizing the nanomultilayered structure, the interfacial compressive or tensile stress can be imposed on the different nanofilms and the influence of the interfacial compressive or tensile stress on the martensitic transformation and even other phase transformations of nanofilms can be experimentally investigated. Therefore, the method of imposing and modulating the interfacial stress through the epitaxial growth structure in the nanomultilayered films should also be noticed and utilized.

## Conclusions

In summary, FeNi/V nanomultilayered films with different V layer thicknesses were synthesized by magnetron sputtering. By adjusting the thickness of the V layer, different interfacial compressive stress were imposed on FeNi layers and the effect of interfacial stress on martensitic transformation in the FeNi film was investigated. Without insertion of V layers, the FeNi film exhibits a fcc structure. With the thickness of V inserted layers up to 1.5 nm, under the coherent growth structure in FeNi/V nanomultilayered films, FeNi layers bear interfacial compressive stress due to the larger lattice parameter relative to V, which induces the martensitic transformation of the FeNi film. As the V layer thickness increases to 2.0 nm, V layers cannot keep the coherent growth structure with FeNi layers, leading to the disappearance of interfacial stress and termination of the martensitic transformation in FeNi films. This investigation verifies that the martensitic transformation could be induced by the nanoscaled interfacial stress in the FeNi nanofilms. The method of imposing and modulating the interfacial stress through the epitaxial growth structure in the nanomultilayered films should also be especially noticed and utilized.

## Competing interests

The authors declare that they have no competing interests.

## Authors’ contributions

WL designed the experiment and wrote the article. PL, KZ, and FM carried out the synthesis of the monolithic FeNi film and FeNi/V nanomultilayered films. XL, XC, and DH assisted in the technical support for measurements (XRD and HRTEM) as well as the data analysis. All authors read and approved the final manuscript.
